# Sex Estimation Based on the Cranial Base of Three-Dimensional Skull Models from the Bosnia and Herzegovina Population Using Geometric Morphometrics

**DOI:** 10.3390/jimaging12070322

**Published:** 2026-07-16

**Authors:** Zurifa Ajanović, Saleha Redžepi, Uzeir Ajanović, Naida Spahović, Amina Zorlak-Čavčić, Emina Dervišević, Admir Terzić, Mirza Pojskić

**Affiliations:** 1 Department of Human Anatomy, Faculty of Medicine, University of Sarajevo, 71000 Sarajevo, Bosnia and Herzegovina; zurifa.ajanovic@mf.unsa.ba; 2 Department of Radiology, Clinical Center University of Sarajevo, 71000 Sarajevo, Bosnia and Herzegovina; saleharedzepi98@gmail.com (S.R.); naidakulenovic@gmail.com (N.S.); admir.terzic.86@gmail.com (A.T.); 3 Department of Information Technologies, Faculty of Engineering, Natural and Medical Sciences, International Burch University, 71000 Sarajevo, Bosnia and Herzegovina; uzeir.ajanovic@gmail.com; 4 Department of Forensic Medicine, Faculty of Medicine, University of Sarajevo, 71000 Sarajevo, Bosnia and Herzegovina; amina.zorlak@mf.unsa.ba (A.Z.-Č.);; 5 Department of Neurosurgery, University Hospital Marburg, Philipps University Marburg, 35037 Marburg, Germany

**Keywords:** sex estimation, cranial base, geometric morphometrics, 3D skull models

## Abstract

Sex estimation is a fundamental component of biological profiling in forensic anthropology, particularly when skeletal remains are incomplete or fragmented. This study aimed to evaluate sex estimation of the cranial base using geometric morphometrics and to assess the predictive value of cranial base morphology for sex estimation. The study included 211 adult skulls (139 male, 72 female) from the Bosnian population. Each skull was digitized to generate 3D models, and 27 anatomical landmarks were recorded. Landmark coordinates were standardized using Generalized Procrustes Analysis, Principal Component Analysis, Discriminant Function Analysis with permutation testing, and regression of shape on centroid size. Statistically significant sex estimation was observed at both the form (shape and size) and shape levels. Classification accuracy based on cranial base form reached 92.81% for males and 86.11% for females. Shape-based classification, after removal of size effects, also showed high accuracy (90.65% for males and 81.94% for females). Regression analysis indicated that size contributed significantly but modestly to shape variation. The cranial base exhibits stable sexually dimorphic patterns and may represent a reliable anatomical region for sex estimation. These findings contribute to population-specific standards for the Bosnia and Herzegovina population and support the forensic applicability of 3D geometric morphometric approaches.

## 1. Introduction

Sexual dimorphism is defined as systematic differences in size and morphology between males and females of the same species. It is expressed across numerous anatomical regions, including the skeletal system. As a result, skeletal remains have long served as a reliable basis for sex estimation in bioanthropological and forensic investigations [1]. In practical forensic contexts, sex assessment is a fundamental component of constructing the biological profile of unidentified human remains. It is routinely applied in archaeology, forensic medicine, criminal investigations, and forensic anthropology [2,3].

A primary objective of forensic anthropology is the reliable identification of human skeletal remains through reconstruction of the biological profile, which commonly includes sex, age, ancestry, and stature estimation. Among these parameters, sex estimation plays a particularly significant role. Determining biological sex substantially narrows the identification process in medico-legal investigations involving decomposed, burned, or skeletonized remains. Conventional skeletal approaches to sex estimation are generally categorized into qualitative (morphological) and quantitative (metric) methodologies [4].

Qualitative methods rely primarily on the visual evaluation of morphological traits, including skeletal robustness, the prominence of muscular attachment sites, and the development of osseous ridges [1]. In contrast, quantitative approaches are based on standardized measurements and statistical procedures, including discriminant function analysis [5]. Although morphological assessment allows relatively rapid examination, its accuracy may be influenced by observer subjectivity and professional experience. Osteometric and statistically based methods provide greater objectivity, reproducibility, and analytical consistency, which explains their increasing application in forensic anthropology [1,6].

The pelvis is widely regarded as the most reliable skeletal region for sex estimation; however, in forensic practice it is frequently unavailable, incomplete, or fragmented [7]. Under such circumstances, the skull represents an important alternative source of diagnostic information. Numerous cranial regions exhibit measurable sexually dimorphic characteristics related to both size and morphology [8]. Craniofacial variation reflects the interaction of multiple biological and functional factors, including muscular development, respiratory demands, metabolic activity, and hormonal influences [5]. Due to its relative structural compactness and high preservation potential, the cranium plays a central role in forensic identification, either through examination of the entire skull or specific anatomical regions [3,9].

Beyond its value in sex estimation, the craniofacial skeleton also contains morphological features relevant for the assessment of population affinity and ancestry, further increasing its significance in biological and forensic profiling [10]. Nevertheless, skeletal morphology demonstrates considerable population variability. Genetic background, environmental influences, nutritional status, socioeconomic conditions, migration patterns, and long-term demographic processes all contribute to observed morphological diversity. Consequently, standards developed for one population cannot always be reliably applied to another, highlighting the need for population-specific sex estimation models [1].

The degree and expression of cranial sexual dimorphism vary among human populations as a result of complex interactions between genetic inheritance, environmental adaptation, climatic conditions, nutrition, and socioeconomic influences [5]. Therefore, the development of population-specific forensic standards remains essential for achieving accurate and reliable sex estimation [1]. Within the cranial complex, the cranial base represents a particularly informative region for investigating sex estimation [5]. It comprises several anatomically and functionally important structures, including the foramen magnum region, occipital condyles, mastoid portion, and adjacent muscular attachment areas. These structures have been shown to reflect sex-related differences in cranial morphology, robustness, and structural organization [8].

The cranial base possesses several characteristics that increase its value in forensic investigations. Its compact morphology, protected anatomical location, and resistance to physical damage contribute to a high likelihood of preservation. As a result, cranial base structures are often retained even in fragmented, commingled, or thermally altered skeletal remains [5]. This preservation potential is particularly important in medico-legal investigations where more fragile cranial regions may be damaged or absent. Recent studies have highlighted the forensic importance of the cranial fossae because their protected intracranial position and relatively thick osseous architecture increase the likelihood of survival under adverse forensic and taphonomic conditions [4].

Contemporary anthropological and forensic research increasingly incorporates digital imaging and three-dimensional analytical techniques for the precise quantification of morphological variation. Advances in computed tomography (CT), multi-slice computed tomography (MSCT), and virtual anthropology have substantially expanded the possibilities of forensic skeletal analysis. Three-dimensional imaging allows high-resolution visualization and non-destructive examination of anatomically complex skeletal regions while improving the reproducibility of morphometric analyses [10,11,12,13,14,15].

Among these approaches, geometric morphometrics (GM) has emerged as a particularly influential methodological framework. Unlike traditional morphometric methods, GM characterizes biological form through configurations of geometric coordinates that remain comparable after the removal of translational, rotational, and scaling effects [2]. This procedure produces a standardized representation of shape while preserving spatial relationships among anatomical landmarks. The methodological basis of GM is the Generalized Procrustes Analysis (GPA), a landmark-based alignment procedure that enables direct comparison of shapes across specimens [12]. The integration of CT-derived three-dimensional reconstructions with geometric morphometric techniques has significantly improved the precision and statistical robustness of forensic anthropological investigations [16]. Such advances are especially valuable for anatomically complex cranial regions that are difficult to evaluate using conventional morphoscopic approaches.

Geometric morphometrics provides a robust framework for the quantitative analysis of biological form through landmark-based shape assessment. Anatomical landmarks are placed on images or three-dimensional models to capture the geometry of morphological structures while preserving spatial information for subsequent statistical analyses [2]. A major advantage of this approach is the clear separation of shape and size, with size commonly expressed as centroid size, defined as the square root of the summed squared distances of all landmarks from their centroid. This framework also enables the use of landmarks and semi-landmarks to reconstruct complex anatomical structures in three dimensions, thereby improving the accuracy of morphological assessment [17].

Traditional morphometric approaches rely primarily on linear measurements and may be affected by differences in size and orientation among specimens. Geometric morphometrics overcomes these limitations by generating a standardized coordinate-based representation of shape suitable for advanced multivariate statistical analyses [18].

Geometric morphometric techniques have been widely applied in studies of cranial variation and forensic sex estimation, providing valuable insights into patterns of sexual dimorphism in different cranial regions. Previous studies have demonstrated that analyses of cranial base structures and the hard palate can achieve high classification accuracy and may provide useful alternatives when traditionally preferred skeletal elements are unavailable [19,20,21,22].

In parallel with the development of geometric morphometric methodologies, statistical procedures such as discriminant function analysis and logistic regression have become increasingly incorporated into forensic sex estimation research [22,23]. These approaches improve predictive performance while simultaneously allowing estimation of classification probabilities and model reliability. Furthermore, regional investigations conducted on osteological material from Bosnia and Herzegovina have already demonstrated the applicability of 3D GM approaches in the analysis of the foramen magnum region, thereby providing both methodological and population-specific foundations for further cranial base research [23].

Given the growing body of evidence demonstrating that cranial base morphology reflects biologically meaningful patterns of sexual dimorphism, further investigation of this region is important not only for improving sex estimation but also for advancing our understanding of population-specific cranial variation. As shown in Table 1, previous studies have reported varying levels of accuracy for sex estimation based on the foramen magnum, occipital condyles, and cranial base across different populations, highlighting both the forensic relevance of these structures and the influence of population-specific morphological variation. However, despite the growing number of studies in this field, no study has yet applied geometric morphometric analysis to the entire cranial base in the population of Bosnia and Herzegovina, highlighting a significant gap in the current literature and justifying the need for the present research. In forensic practice, the cranial base may represent one of the few preserved anatomical regions available for examination in cases involving fragmented, burned, or otherwise compromised human remains. Therefore, the evaluation of cranial base morphology in the population of Bosnia and Herzegovina has both biological and practical significance. Beyond classification performance, such analyses contribute to the development of population-specific forensic standards and provide additional tools for sex estimation when traditionally preferred skeletal elements are unavailable, thereby strengthening the reliability of biological profile reconstruction in real forensic casework.

The specific objectives of the study are as follows:

1. To determine whether statistically significant sex-related differences exist in cranial base form (shape combined with size) using geometric morphometric analyses and discriminant statistical procedures.
2. To assess sex-related differences in cranial base shape after controlling for the influence of size, thereby eliminating potential allometric effects.
3. To quantify the contribution of size, expressed as centroid size, to overall cranial base shape variability.
4. To calculate the classification accuracy of sex estimation models derived from the obtained morphometric data, with the aim of evaluating the practical applicability of the method in forensic anthropological contexts.

Research Hypotheses:

**H1**  (Primary hypothesis)**.** *The cranial base in three-dimensional skull models of the Bosnia and Herzegovina population demonstrates statistically significant sexual dimorphism in form (shape combined with size), enabling accurate sex estimation through the application of geometric morphometric techniques and MorphoJ-based analytical procedures.*

**H2.**  *After controlling for size effects (centroid size), statistically significant sex-related differences in the shape of the cranial base remain detectable.*

**H3.**  *The size of the cranial base, expressed as centroid size, contributes significantly to shape variability (reflecting an allometric influence), but does not fully account for the observed sex-related differences in cranial base morphology. The null hypotheses are the opposite of the research hypotheses.*

Anatomical justification for each landmark is provided in Table 2, which includes detailed definitions and references to previously published standards.

The remainder of this article is organized as follows. Section 2 describes the study sample, data acquisition procedures, landmark definition, and the geometric morphometric methods applied in the analysis. Section 3 presents and discusses the results of cranial base shape variation and sex estimation. The final section provides the main conclusions, highlights the forensic implications of the findings, and outlines directions for future research.

## 2. Materials and Methods

The principal objective of this investigation is to examine sexual dimorphism of the cranial base in three-dimensional (3D) models of human skulls from the population of Bosnia and Herzegovina using a geometric morphometric approach implemented in the MorphoJ software package (version 1.07a; developed by Chris P. Klingenberg, Manchester, UK), as well as to evaluate the predictive performance of sex estimation models derived from cranial base morphology, considering both shape and form [28].

The study was conducted as a prospective geometric morphometric investigation at the Department of Human Anatomy, Faculty of Medicine, University of Sarajevo. The sample consisted of 211 macerated and degreased adult skulls of known sex and age, including 139 males and 72 females. All specimens originated from the Osteological Collection of the same institution and date to the mid-twentieth century, corresponding to the period surrounding the Second World War, representing individuals from the Bosnia and Herzegovina population.

Only anatomically well-preserved skulls without evident pathological alterations or structural damage were included, while damaged specimens were excluded from the analysis. The known sex of all skulls was used as a control variable for subsequent discriminant statistical analyses and model validation.

Each skull was digitized using a laser-based HP 3D Structured Light Scanner Pro S2 (DAVID SLS-2; HP Inc., Palo Alto, CA, USA), producing high-resolution three-dimensional cranial models. The resulting digital reconstructions were processed and standardized into formats compatible with morphometric software, enabling accurate landmark identification and export of coordinate data for further statistical analysis.

For cranial base analysis, a configuration of 27 anatomical landmarks was defined, including 12 paired (bilateral) and 3 unpaired (midline) landmarks. All landmarks were identified and digitized using Landmark Editor software (version 3.6) with careful placement at homologous anatomical positions across all specimens to ensure consistency. A complete list of anatomical landmarks, together with their definitions and supporting references, is provided in Table 2, following previously established geometric morphometric protocols [21].

Landmark acquisition was performed on standardized three-dimensional cranial models using Landmark Editor software. Each landmark was placed manually on homologous anatomical points defined according to established anthropological and osteological criteria. Landmarks were selected based on their repeatability, clear anatomical definition, and presence across all specimens. Prior to digitization, a consistent protocol was applied to ensure uniform orientation of each 3D model. Landmarks were recorded in a fixed sequence to minimize operator variability, and all points were checked for consistency across specimens before export.

Landmark coordinates were exported in NTSYS format and imported into MorphoJ software for subsequent geometric morphometric analyses.

Geometric morphometrics provides a coordinate-based framework for quantifying biological shape while preserving spatial relationships among landmarks after removal of non-shape variation. Generalized Procrustes Analysis (GPA) was applied to eliminate differences in size, position, and orientation by scaling configurations to unit centroid size, translating them to a common origin, and rotating them to minimize inter-landmark distances. Generalized Procrustes Analysis (GPA) was performed following established geometric morphometric procedures [14,15,29].

All statistical analyses were performed in MorphoJ, including Generalized Procrustes Analysis (GPA), Principal Component Analysis (PCA), Discriminant Function Analysis (DFA), and regression analysis. Permutation tests (1000 iterations) were used to assess statistical significance, with a significance level set at *p* < 0.05. A leave-one-out cross-validation procedure was applied to evaluate the robustness of the DFA classification results.

Regression of shape on centroid size was conducted to quantify allometric effects and to separate size-related from shape-related sexual dimorphism. Centroid size was extracted from the Procrustes-aligned configurations and used as a proxy for overall cranial size in subsequent allometric analyses.

**Figure 1:**
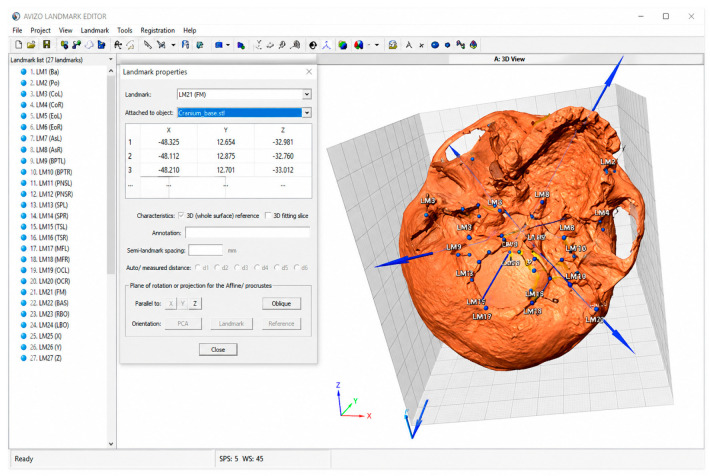
Landmarks marked on the cranial base.

**Figure 2:**
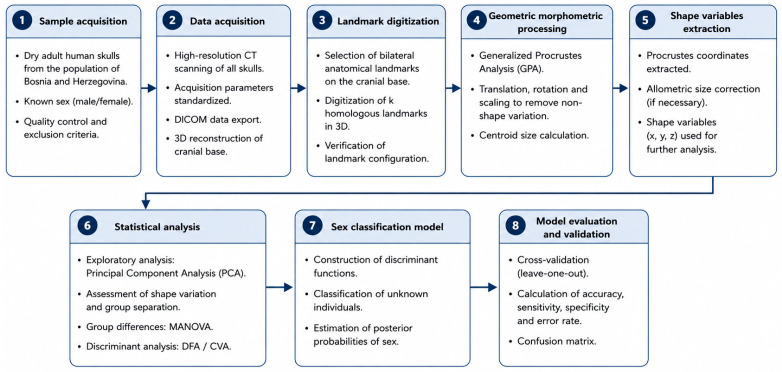
Schematic overview of the methodological workflow. The analysis comprised eight main steps from data acquisition to model validation, including geometric morphometric processing and statistical evaluation for sex estimation.

An overview of the landmark configuration workflow used in the study is provided in Figure 1 and a schematic overview of the methodological workflow is presented in Figure 2.

## 3. Results and Discussion

**Figure 3:**
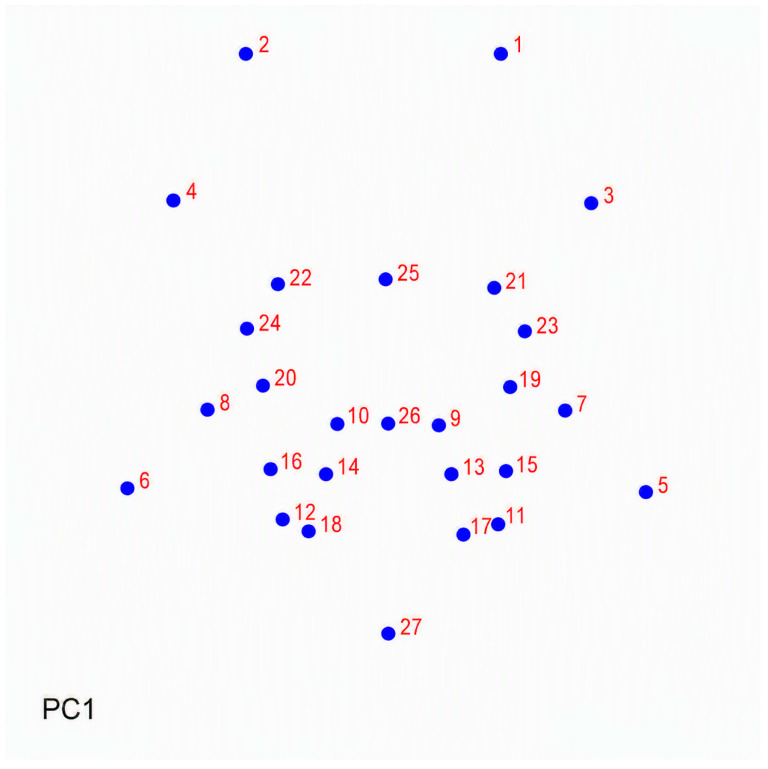
Shape variation patterns of the cranial base described by Principal Component 1 Blue circles represent the mean positions of anatomical landmarks. 1 i 2-infraorbitale, 3 i 4-infratemporale, 5 i 6-mastoidale, 7 i 8-basostyloidion anterior, 9 i 10-occipitocondylion anterior, 11 i 12-occipitocondylion posterior, 13 i 14-occipitocondylion mediale, 15 i 16-occipitocondylion laterale, 17 i 18-foraminolaterale, 19 i 20-caroticum mediale, 21 i 22-ovale mediale, 23 i 24-spinale, 25-hormion, 26-basion, 27-opisthion.

For the analysis of sexual dimorphism of the cranial base, a total of 27 anatomical (anthropometric) landmarks including 12 paired and 3 unpaired points were identified and digitized using the Landmark Editor software on 211 skulls included in the study sample (139 male and 72 female specimens). Patterns of cranial base shape variation were visualized using deformation vectors relative to the mean landmark configuration (Figure 3).

Figure 3 illustrates the corresponding landmark configuration, where shape variability is predominantly expressed in the peripheral regions of the cranial base, particularly along the mastoid–occipital complex. Central cranial base landmarks exhibit markedly lower displacement, indicating greater morphological stability in these areas. This pattern further supports the interpretation that the primary source of variation captured by PC1 is driven by differential expansion and angular modification of the posterolateral cranial base rather than uniform scaling or global size effects.

**Figure 4:**
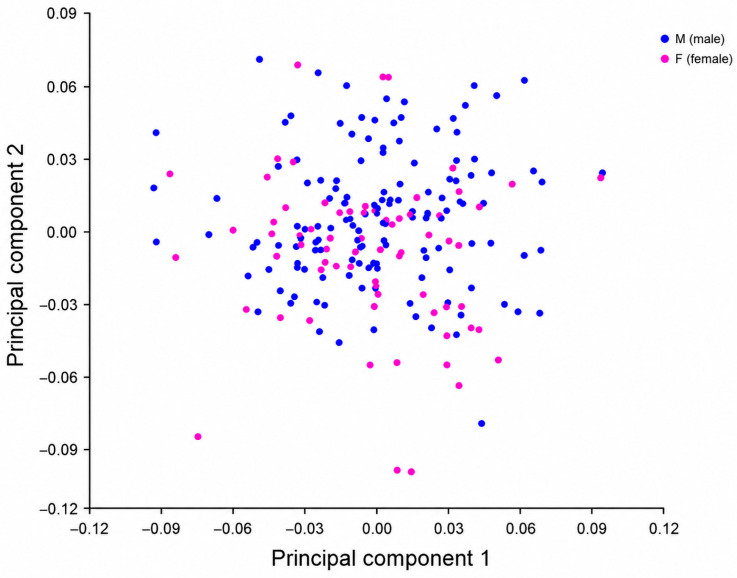
Distribution of skulls from the analyzed sample within the morphospace defined by the first two PCs, based on variation in cranial base form (shape and size). M (blue points)—males, F (pink points)—females. The overlapping areas simply indicate observations falling within the same value ranges for both groups and do not obscure the comparison between male and female distributions.

Following Procrustes superimposition, Procrustes distances between groups were calculated in MorphoJ software. Sex was introduced as a grouping variable for the analysis of differences in both form (shape + size) and shape of the cranial base. Principal Component Analysis (PCA) was subsequently performed based on the covariance matrix. The PCA results indicated that the first two principal components (PC1 and PC2) together accounted for 26.687% of the total variability (Table 3). The distribution of individual specimens within the morphospace defined by the first two principal components is presented in Figure 4.

Figure 4 demonstrates a substantial overlap between male and female specimens within the morphospace defined by PC1 and PC2. Nevertheless, a tendency toward partial separation of the two sex groups was observed, indicating the presence of sex-related variation in cranial base form. The overlap between groups suggests that individual variation remains considerable and that cranial base form alone does not completely distinguish male from female specimens. PC1 and PC2 accounted for 14.56% and 12.12% of the total variation, respectively, together describing 26.69% of overall cranial base form variability.

To determine whether statistically significant sex-related differences exist in the shape and size (form) of the cranial base, a Discriminant Function Analysis (DFA) with classification testing was performed. The calculated Procrustes distance between group means was 0.0306. The permutation test with 1000 iterations yielded a *p*-value < 0.0001, indicating a statistically significant sex-related difference in cranial base form. The classification results showed that, of the 139 male skulls, 129 were correctly classified as male, corresponding to an accuracy of 92.81%. Among the 72 female skulls, 62 were correctly classified as female, yielding a classification accuracy of 86.11% (Table 4). The overall classification accuracy of the model was 90.5%.

**Figure 5:**
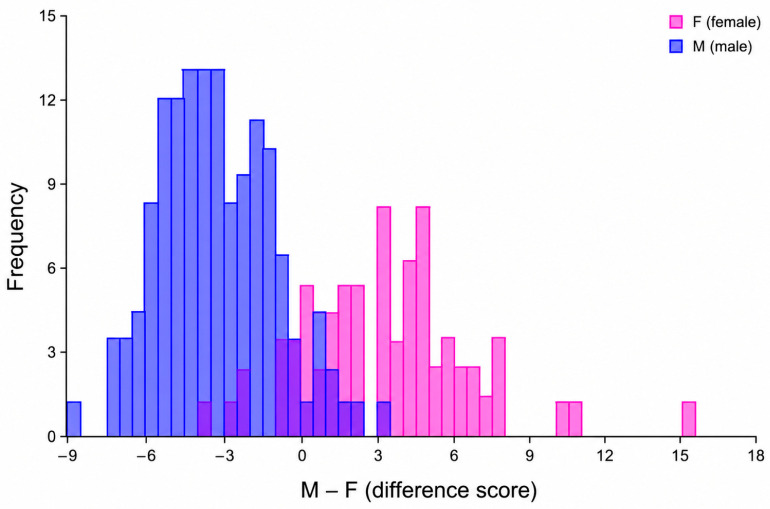
Discriminant function analysis of the influence of cranial base shape and size on sexual dimorphism. M—male, F—female. The overlapping areas simply indicate observations falling within the same value ranges for both groups and do not obscure the comparison between male and female distributions.

The results of the Discriminant Function Analysis (DFA) examining the influence of cranial base shape and size on sexual dimorphism in the analyzed skull sample are presented in Figure 5.

In contrast to PCA, which explores overall morphological variation, DFA maximized differences between predefined sex groups. The distribution of specimens along the discriminant axis demonstrated a clearer separation between males and females, although some overlap remained. This finding is consistent with the classification results, which showed a high overall accuracy of 90.5%, indicating that cranial base form contains substantial sexually dimorphic information despite the overlap observed in the PCA morphospace.

**Figure 6:**
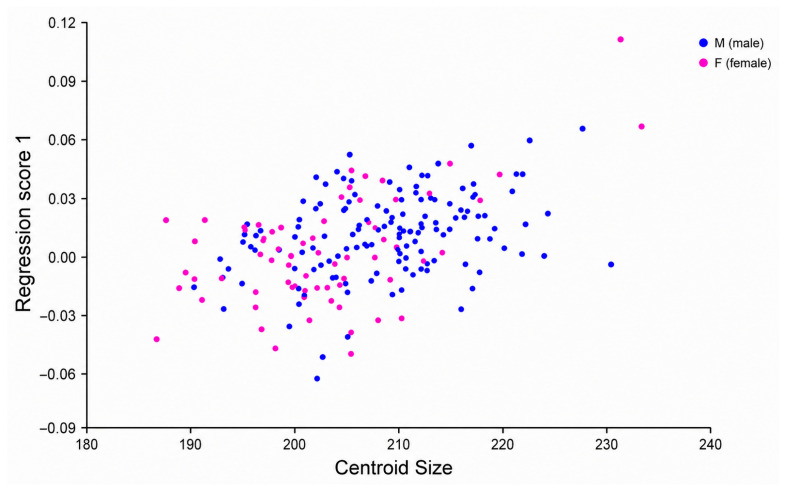
Influence of cranial base size on sexual dimorphism of cranial base shape. M (blue points)—males, F (pink points)—females. The overlapping areas simply indicate observations falling within the same value ranges for both groups and do not obscure the comparison between male and female distributions.

Size was represented by centroid size as the primary metric of overall structural dimensions. The regression results indicated that size accounted for 1.729% of the total shape variability of the cranial base. The relationship was statistically significant (*p* < 0.0001, permutation test with 10,000 iterations). The effect of cranial size and the distribution of specimens within the morphospace as a function of cranial base size are illustrated in Figure 6.

Regression analysis demonstrated that centroid size contributed significantly to cranial base variation, although the proportion of explained shape variance was relatively small (1.729%). The distribution of specimens suggests that size contributes to sexual dimorphism but does not fully account for the observed differences in cranial base morphology.

**Figure 7:**
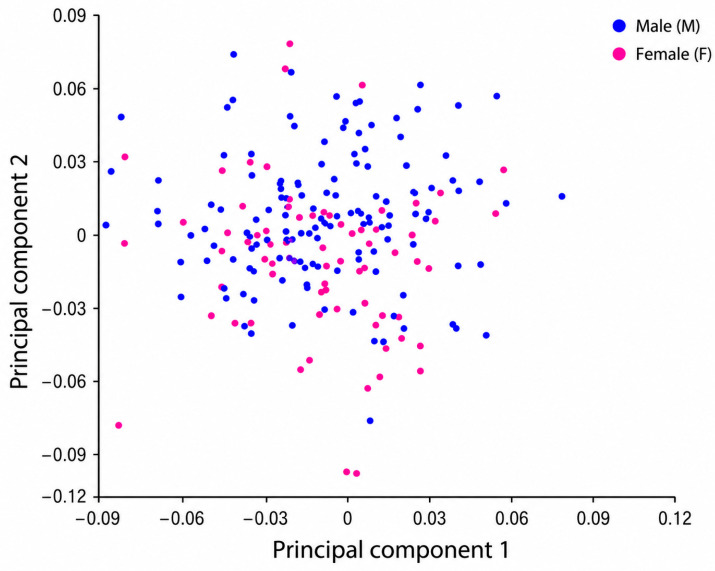
Distribution of skulls from the analyzed sample within the morphospace defined by the first two PCs, based on variation in cranial base shape. M (blue points)—males, F (pink pints)—females. The overlapping areas simply indicate observations falling within the same value ranges for both groups and do not obscure the comparison between male and female distributions.

After removal of size-related effects, the PCA scatterplot continued to show partial separation between male and female specimens, accompanied by substantial overlap between groups (Figure 6). The persistence of this pattern indicates that sexual dimorphism of the cranial base is not exclusively attributable to differences in size but also reflects shape variation independent of allometric effects. PC1 and PC2 accounted for 14.30% and 12.23% of total shape variation, respectively. The first two principal components accounted for 26.528% of the total shape variability of the cranial base (Table 5), and the distribution of specimens within the corresponding morphospace is presented in Figure 7.

To evaluate sex-related differences in cranial base shape independent of size, Discriminant Function Analysis (DFA) with permutation testing was performed. The Procrustes distance between group mean configurations was 0.0292, and the permutation test with 1000 iterations yielded *p* < 0.0001, indicating a statistically significant sex-related difference in cranial base shape independent of size effects.

The classification matrix (Table 6) showed that, of the 139 male skulls, 126 were correctly classified as male (accuracy 90.65%), whereas 13 were misclassified as female. Among the 72 female skulls, 59 were correctly classified (accuracy 81.94%), while 13 were incorrectly assigned to the male group. The overall classification accuracy after removal of size effects was 87.7%.

**Figure 8:**
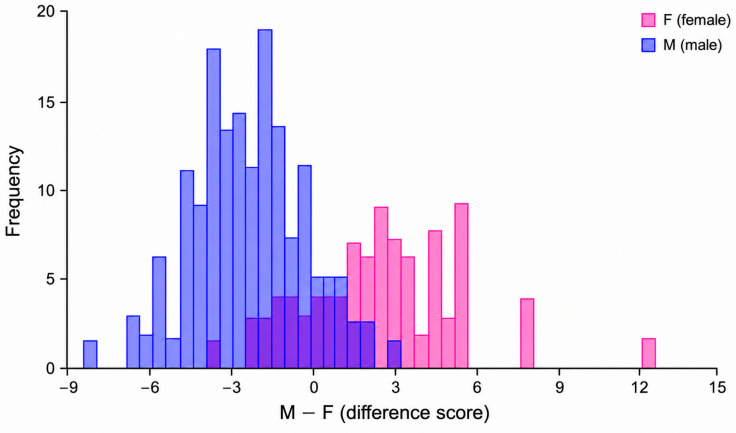
Discriminant function analysis of the influence of cranial base shape on sexual dimorphism. M—male, F—female. The overlapping areas simply indicate observations falling within the same value ranges for both groups and do not obscure the comparison between male and female distributions.

**Figure 9:**
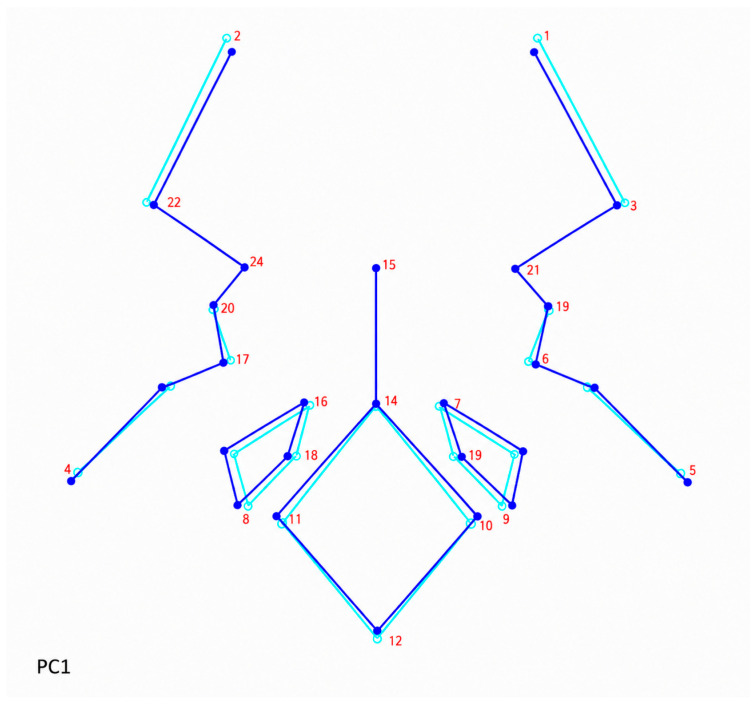
Range of cranial base shape variation in the analyzed skull sample.

The results of the Discriminant Function Analysis (DFA) of cranial base shape after removal of size effects are presented in Figure 8, while the range of shape variation (deformation visualization) is illustrated in Figure 9.

The DFA performed on shape variables after removal of size effects demonstrated a separation between male and female specimens that was less pronounced than that observed for form (shape and size combined). This finding is reflected in the slightly lower classification accuracy (87.7%) and suggests that size contributes to the discriminatory power of the model, although shape alone remains significantly associated with sex.

In Figure 9, the wireframe deformation associated with PC1 demonstrates that the most pronounced shape changes are concentrated in the posterolateral and posterior basal regions of the cranial base. The greatest deformations, visualized by the displacement and divergence of connecting wireframe lines between corresponding landmarks, are primarily located in the mastoid region of the temporal bone and extend posteriorly toward the occipital base. In these areas, an outward and slightly superior displacement of landmarks is evident, indicating localized expansion and angular reconfiguration of the posterolateral cranial base. Additional contributions to this pattern are observed in the region of the occipital condyles and the adjacent basilar portion of the occipital bone, which together reflect posterior basal remodeling captured by PC1. In contrast, landmarks that appear as relatively stable points with minimal vector displacement show limited contribution to the overall shape variation. These regions remain comparatively conserved across specimens, suggesting lower morphological variability and reduced involvement in the primary axis of shape change described by PC1.

Taken together, Figure 3 and Figure 9 consistently demonstrate that PC1 represents biologically meaningful shape variation localized within the mastoid and occipital components of the cranial base, reflecting region-specific morphological remodeling rather than diffuse or homogeneous shape change.

The cranial base was selected as the focus of this study due to its unique anatomical and developmental characteristics that make it particularly valuable in forensic anthropological contexts. Compared to other cranial regions commonly used for sex estimation, such as the facial skeleton or the pelvis, the cranial base is less influenced by postnatal environmental factors and exhibits relatively late ontogenetic fusion, contributing to its structural stability throughout adulthood. In forensic scenarios involving fragmented, commingled, or partially preserved skeletal remains, the cranial base is frequently preserved owing to its protected anatomical position at the skull’s internal foundation [8].

This is particularly relevant in forensic casework where cranial fragments are recovered in isolation, and where traditional sex estimation methods based on the pelvis or facial skeleton cannot be applied. While the pelvis remains the most reliable region for sex estimation due to its high degree of sexual dimorphism, it is often absent or severely damaged in forensic casework, and the facial skeleton, although informative, is more susceptible to environmental, functional, and population-specific variation. However, the cranial base generally exhibits more subtle sexual dimorphism compared to the pelvis, and its relatively smooth morphology with fewer clearly defined anatomical landmarks may limit the application of traditional morphometric approaches. These limitations justify the use of three-dimensional geometric morphometrics, which enables precise quantification of complex shape variation and allows the extraction of biologically meaningful information even from anatomically less discrete regions. Together, these properties make the cranial base a relevant but methodologically challenging region, suitable for further investigation in population-specific sex estimation models [1].

The findings demonstrated statistically significant sex-related differences at both the form level (shape combined with size) and the shape level after correction for size effects. This indicates that cranial base morphology contains a strong sexually dimorphic signal that may be effectively applied in forensic identification, particularly in cases where more diagnostic skeletal regions, such as the pelvis, are unavailable or poorly preserved [29,30].

The classification performance obtained in the present study is broadly consistent with previous findings on cranial sexual dimorphism using geometric morphometric approaches. The first principal components were interpreted as reflecting global cranial base shape variation characterized by coordinated morphological changes across the posterolateral and posterior basal regions. The most pronounced deformations were observed in the mastoid region of the temporal bone (pars mastoidea ossis temporalis/processus mastoideus), where lateral expansion and superior displacement of landmarks were evident. Additional variation was detected in the occipital base, particularly involving the occipital condyles (condyli occipitales) and the basilar part of the occipital bone (pars basilaris ossis occipitalis), indicating integrated posterior basal remodeling. These findings suggest that the primary axis of variation is driven by regionally coordinated shape changes within the mastoid–occipital complex rather than isolated localized deformation patterns.

Similar levels of discriminatory power have been reported in analyses of cranial base morphology, where Chovalopoulou and colleagues demonstrated that three-dimensional landmark-based models can achieve statistically significant but moderate sex classification accuracy, reflecting the relatively subtle expression of sexual dimorphism in this anatomical region. Comparable observations were also made by Bigoni and colleagues, who noted that while the cranial base exhibits measurable sexual differences, its discriminative capacity is generally lower than that of more dimorphic skeletal regions such as the pelvis or facial skeleton [22].

In agreement with the broader morphometric framework proposed by Bookstein, the present findings further support the importance of allometric effects in shaping cranial sexual dimorphism. Studies applying geometric morphometrics consistently show that models incorporating both shape and size (form) tend to outperform shape-only analyses, as centroid size captures an important component of sexual variation. This pattern is also reflected in the current results, where the inclusion of size contributed to improved classification accuracy compared with shape-only models [13].

Overall, differences in reported classification performance across studies are likely related to methodological variation, population-specific cranial morphology, and differences in landmark configurations and analytical strategies. These factors highlight the importance of interpreting discriminant results within both methodological and biological contexts rather than as absolute values.

Allometric evaluation indicated that cranial base size had a statistically significant but quantitatively modest influence on shape variability, suggesting that sexual differences cannot be explained solely by overall size variation but also involve intrinsic shape-related components. Previous geometric morphometric research has demonstrated that cranial shape variation is strongly structured by size-related allometric effects. This is particularly evident along the first principal component, where scaling effects may represent a dominant source of morphological variation, while subsequent components tend to reflect shape variation that is less strongly associated with overall size. These findings are consistent with the general geometric morphometric framework, which treats sexual dimorphism as a combination of size-dependent and size-independent components of form variation, and allows formal partitioning of these effects through multivariate statistical approaches [31]. The persistence of significant differences after size adjustment further supports the presence of stable dimorphic morphological patterns independent of size effects.

Due to the limited availability of discrete landmarks on smooth cranial base surfaces, most geometric morphometric analyses of the skull focus on regions such as the face and cranial base, where semilandmarks are commonly applied to capture continuous anatomical curvature [32]. Semilandmarks (sliding landmarks), first introduced by Bookstein (1991) and later developed for three-dimensional applications, allow points to slide along tangent directions to minimize shape differences while preserving biologically meaningful variation perpendicular to the surface [13]. This approach enables more accurate representation of complex cranial base morphology.

Previous investigations have reported variable success rates in sex estimation based on cranial base morphology, highlighting the importance of both methodological design and population characteristics. The skull base is a complex bony structure forming the interface between the brain and extracranial anatomy, and it contains multiple foramina and neurovascular passageways that contribute to high morphological variability. Because of this structural complexity, morphometric studies have often focused on specific regions such as the clivus, sella turcica, and foramina, each of which demonstrates considerable inter-individual and inter-population variation [33]. For example, Bigoni et al., studying a Central European sample, identified pronounced craniofacial dimorphism; however, the cranial base did not consistently provide a strong discriminatory signal in their analysis. Differences between studies may reflect variation in landmark selection, anatomical coverage of the analyzed region, as well as demographic and population-related factors such as ancestry, historical period, age structure, and sex distribution [21].

Research conducted on a Greek population by Chovalopoulou et al. demonstrated that sex estimation based on cranial base shape alone achieved lower accuracy than analyses incorporating both shape and size, emphasizing the contribution of size-related information to classification performance. Such findings are consistent with the general expectation in geometric morphometrics that inclusion of size variables frequently enhances discriminative power because part of sexual variation is associated with size dimorphism. Differences in classification performance between studies may therefore be attributed to population-specific morphology, variations in landmark configurations, validation strategies, and sample composition [22].

Skeletal morphology, including cranial structures, is known to exhibit pronounced population variability, meaning that models derived from one population cannot necessarily be applied directly to another. Therefore, while the present discriminant functions demonstrate strong population-specific performance, their direct applicability to other populations is limited due to known inter-population variation in cranial morphology. In this context, the present results contribute to the development of population-specific standards for sex estimation in the Bosnia and Herzegovina population, which is of particular importance for local forensic practice. Additional support for the relevance of cranial base morphology in sex estimation is provided by earlier regional investigations focusing on the foramen magnum, including both traditional craniometric and more recent three-dimensional geometric morphometric approaches, which have demonstrated the diagnostic potential of this anatomical region [34].

Several limitations should be acknowledged. The sex distribution of the sample was not balanced, which may influence classification stability and the pattern of misclassification between groups. Furthermore, the analyzed material originates from a specific historical period, and the results should therefore be interpreted primarily within the context of the studied population. In addition, intra- and inter-observer landmarking error was not formally quantified, although landmark placement precision is known to affect multivariate shape analyses and classification outcomes. Future studies should therefore include reproducibility assessments on subsamples, as well as additional validation procedures, particularly when models are intended for forensic casework applications.

Additionally, no formal a priori sample size or statistical power analysis was performed to determine whether the study was optimally powered for detecting subtle patterns of sex estimation. The selection and spatial distribution of landmarks may also introduce methodological constraints, particularly in anatomically smooth regions such as the cranial base where the number of clearly homologous landmarks is limited. This may reduce the ability of the configuration to fully capture complex shape variation. Furthermore, multivariate classification approaches such as discriminant function analysis are inherently sensitive to sample composition and may be affected by potential overfitting in the absence of independent external validation. Finally, the absence of validation on independent populations limits the generalizability of the derived models beyond the Bosnian and Herzegovinian sample.

Age-related morphological variation may also represent a potential source of variation in cranial base shape; however, all specimens in the present study were adult individuals, which reduces but does not completely eliminate the potential influence of age on the observed morphological patterns. While external independent validation would be the most robust approach, it was not feasible due to the limited availability of comparable osteological samples.

Overall, 3D geometric morphometrics of the cranial base represent a methodologically robust and practically valuable approach for sex estimation in the Bosnia and Herzegovina population. Geometric morphometric approaches have increasingly been extended to clinical and forensic applications, where landmark-based methods combined with Procrustes superimposition allow for detailed quantification of cranial shape variation beyond traditional linear measurements. This has improved the ability to detect subtle morphological differences that are otherwise not captured by classical craniometric techniques. The presence of clear dimorphic patterns, together with the stability of shape differences after size correction, indicates that the cranial base constitutes a reliable anatomical region for inclusion in population-specific identification protocols. Some cranial base deformation patterns observed in broader craniofacial research further support the concept that cranial base morphology is integrated with overall craniofacial development, influencing adjacent structures such as the maxilla, orbit, and nasal complex.

These findings provide a foundation for further validation on independent samples and for integration with analyses of additional cranial regions and advanced digital morphometric techniques. They also reinforce the idea that the cranial base functions as a central architectural unit in craniofacial morphology rather than an isolated structure.

## 4. Conclusions

This study demonstrated statistically significant sexual dimorphism of the cranial base in a Bosnia and Herzegovina population using three-dimensional geometric morphometric analysis. Sex estimation based on combined cranial base form (shape and size) achieved high classification accuracy (male: 92.81%, female: 86.11%). When size effects were removed, shape-only analysis still yielded strong discriminatory performance (male: 90.65%, female: 81.94%), indicating that cranial base shape alone carries substantial sexual signal.

Although the influence of size was statistically significant, its overall contribution was quantitatively small (1.729%), suggesting the presence of intrinsic or “pure” shape-related sexual dimorphism independent of size variation.

These findings support the forensic relevance of cranial base morphology for sex estimation and highlight the importance of developing population-specific reference standards. However, the results should be interpreted within a population-dependent framework and cannot be directly generalized beyond the sample studied. Further validation on independent populations, as well as additional assessment of landmarking reliability and methodological robustness, is necessary to confirm the broader applicability of the proposed approach in forensic practice.

## Figures and Tables

**Table 1:** State-of-the-art studies on sexual dimorphism and sex estimation using the cranial base, foramen magnum, and occipital condyle morphology in different populations.

Author(s)	Year	Population	Anatomical Region	Method
Gapert, Black & Last [24]	2009	United Kingdom	Foramen magnum	Discriminant Function Analysis (DFA)
Uysal et al. [25]	2005	Turkish	Foramen magnum and occipital condyles (3D CT)	Discriminant Function Analysis
Edwards et al. [26]	2013	Swiss (Virtopsy CT)	Foramen magnum	Discriminant Function Analysis
Chovalopoulou & Bertsatos [22]	2017	Greek	Cranial base	Geometric morphometrics
Alnemri et al. [27]	2021	Jordanian	Foramen magnum and occipital condyles	Discriminant Function Analysis
Ajanović et al. [23]	2023	Bosnian and Herzegovinian	Foramen magnum (3D geometric morphometrics)	3D Geometric Morphometric Analysis

**Table 2:** Anatomical landmarks used for cranial base analysis with definitions and references (adapted from [21] and standard anatomical nomenclature).

Anthropometric Landmark	Location
Infraorbitale	The most lateral point on the lateral margin of the infraorbital foramen.
Basostyloidion anterior	A point located on the anterior surface of the styloid process.
Foraminolaterale	The most lateral point on the foramen magnum.
Occipitocondylion laterale	The most lateral point on the occipital condyle.
Occipitocondylion mediale	The most medial point on the occipital condyle.
Occipitocondylion anterior	A point on the anterior margin of the occipital condyle.
Occipitocondylion posterior	A point on the posterior margin of the occipital condyle.
Caroticum mediale	The most medial point on the external opening of the carotid canal.
Ovale mediale	The most medial point on the margin of the foramen ovale.
Spinale	The most medial point on the margin of the foramen spinosum.
Mastoidale	The lowest point on the mastoid process.
Infratemporale	The intersection of the sphenosquamosal suture and the infratemporal crest.
Basion	The midpoint of the anterior margin of the foramen magnum.
Opisthion	The midpoint of the posterior margin of the foramen magnum.
Hormion	The intersection of the midsagittal line and the junction of the vomer with the sphenoid bone.

**Table 3:** Eigenvalues and percentage of variability of cranial base form (shape and size) described by eigenvalues obtained from Principal Component Analysis (PCA).

PCs	Eigenvalues	Percentage of Variability %	Cumulative Percentage of Variability %
1.	0.00110	14.56	14.56
2.	0.00091	12.12	26.69
3.	0.00054	7.22	33.90
4.	0.00048	6.39	40.30
5.	0.00046	6.13	46.43
6.	0.00038	5.08	51.51
7.	0.00034	4.55	56.05
8.	0.00033	4.36	60.42
9.	0.00032	4.21	64.62
10.	0.00027	3.57	68.20

**Table 4:** Sex prediction accuracy based on form of skull base.

		Sex Prediction Accuracy		Total
Sex	Male	**129**	10	139
	Female	10	**62**	72
*Total*		*139*	*72*	*211*

**Table 5:** Eigenvalues and percentage of cranial base shape variability described by eigenvalues obtained from Principal Component Analysis (PCA).

PCs	Eigenvalues	Percentage of Variability %	Cumulative Percentage of Variability %
1.	0.00106	14.30	14.30
2.	0.00090	12.23	26.53
3.	0.00054	7.34	33.87
4.	0.00047	6.31	40.18
5.	0.00046	6.23	46.41
6.	0.00036	4.81	51.23
7.	0.00034	4.60	55.83
8.	0.00032	4.35	60.18
9.	0.00032	4.28	64.46
10.	0.00027	3.61	68.06

**Table 6:** Sex prediction accuracy based on the cranial base shape (excluding size effects).

		Sex Prediction Accuracy		Total
Sex	Male	**126**	13	139
	Female	13	**59**	72
*Total*		*139*	*72*	*211*

## Data Availability

The original contributions presented in this study are included in the article. Further inquiries can be directed to the corresponding author.
